# Confusion, communication, and consent: ELSI barriers to genomics-enhanced remediation in Alberta’s oil sands

**DOI:** 10.3389/fgene.2026.1889489

**Published:** 2026-08-07

**Authors:** Michelle Tilford-Shaw, Max Pospisil, Lori Bradford, Graham Strickert, Diane Dupont

**Affiliations:** 1 School of Environment and Sustainability, University of Saskatchewan, Saskatoon, SK, Canada; 2 Ron and Jane Graham School of Professional Development, College of Engineering, University of Saskatchewan, Saskatoon, SK, Canada; 3 Department of Economics, Faculty of Social Sciences, Brock University, St. Catharine’s, ON, Canada

**Keywords:** constructed wetlands, genomics, indigenous engagement, oil sands processed water (OSPW), traditional knowledge

## Abstract

While constructed treatment wetland systems (CTWS) and genomics have been identified as viable remediation options for oil sands processed-affected water, researchers have paid limited attention to the priorities of affected groups. To address this gap, we used focus groups to explore the perspectives on CTWS and genomics held by Indigenous Peoples, oil and gas industry employees, CTWS and genomics researchers, and government regulators and policymakers affected by oil sands remediation in northern Alberta. We identify six key concerns: confusion about the options themselves; challenges with communication about the options; differing perspectives on naturality; differing risk tolerances; questions about long term effects and who will maintain liability; and cultural impacts to Indigenous Peoples. Importantly, participants identified barriers related to ethical, legal, social, environmental, and governance implications, often referred to as ELSI, that affect their participation in decision-making about genomics-enhanced oil sands remediation. We recommend directions for improving meaningful engagement and participation.

## 1 Introduction

Applications of genomics increasingly require attention to ethical, legal, social, environmental, and governance implications, often referred to as ELSI. Greenbaum (2013) frames ELSI as a proactive field that anticipates implications for individuals and society, supports public discussion, and develops policy options for science in social context. Trump et al. (2023) similarly argue that successful biotechnology depends on technical soundness, safety, security, and transparent attention to ELSI throughout technology development and implementation. These imperatives are especially important when genomics is applied to environmental remediation, where technical choices may affect communities, ecosystems, legal responsibilities, public trust, and long-term relationships with land and water. One area where genomics innovations are increasingly important for human thriving is in the realm of remediation of extractive industries, such as in the Canadian oil sands region.

Oil sands mining processes result in large quantities of oil sands process-affected water (OSPW; Quinlan and Tam, 2015; Sandhu et al., 2024), which is stored in tailings ponds that covered about 120 km^2^ of northern Alberta, Canada in 2020 and will grow as mining continues (Environmental Defence Canada and Canadian Parks and Wilderness Society (Environmental Defence Canada and Canadian Parks and Wilderness Society, 2022; Slingerland et al., 2019). Regulations have prohibited the release of OSPW, which contains persistent and toxic naphthenic acid fraction compounds, into waterways (Tanna et al., 2019; Trepanier et al., 2025). However, oil sands development, including the building of extraction site mining pits and tailing ponds, has been based on the premise of returning the site to its initial nature, which includes the ultimate release of remediated OSPW (Tanna et al., 2019).

One remediation technology shown to reduce OSPW contamination is constructed treatment wetland systems (CTWS; Hendrikse et al., 2018; McQueen et al., 2017; Rodgers and Castle, 2008; Trepanier et al., 2025). CTWS are wetlands built as part of a treatment train to reduce wastewater toxicity through natural processes performed by locally- and/or non-locally-occurring plants and microbes. There has already been some pilot use of CTWS in Alberta to investigate its efficacy in treating OSPW (e.g., Cancelli and Gobas, 2020). Research is underway that uses genomics-based methods to understand how wetland plants and microbes degrade and detoxify OSPW, identify the microbes involved in these processes, and enhance the efficacy of CTWS (Muench and Martineau, 2020; Reis et al., 2023; Trepanier et al., 2025). Still, northern Alberta’s harsh climate may affect CTWS enhanced by genomics in ways that are difficult to replicate comprehensively in a controlled setting and require further research (Trepanier et al., 2025).

While Page and Atkinson-Grosjean (2013) found that community groups and First Nations at a mine site in British Columbia expressed reservedly positive attitudes toward ecogenomics-enhanced bioremediation strategies, meaning remediation approaches informed by genomic analysis of organisms and ecological processes, the authors caution that “positive attitudes should not be taken as final acceptance of a technology” (p. 282). However, there has been little similar research with communities affected by the Alberta oil sands. This gap is important for ELSI research because emerging biotechnology applications can face resistance when technical development proceeds without early, transparent attention to risk, safety, governance, and social implications (Trump et al., 2023).

Indigenous and local communities are attentive to environmental concerns with OSPW remediation and release and will be particularly impacted if OSPW remediation and release are not done appropriately (Foote, 2012; Westman and Joly, 2019). Different individuals are affected in diverse ways due to environmental, economic, educational, ethical, legal, social, spiritual, and cultural considerations. Without proper remediation, OSPW threatens water quality and quantity, while harm to wildlife adversely affects subsistence and spiritual practices as well as intergenerational knowledge transfer for Indigenous Peoples (Westman and Joly, 2019). Since Indigenous Peoples are living in areas that will be most impacted by OSPW remediation, their lives are highly affected in all aspects. Policies in Canada emphasize that, for issues like resource development and remediation, Indigenous Peoples must be involved in the decision-making process. Despite these policies, the values and priorities of local communities considering remediation and release have received limited consideration (Carter et al., 2017; Foote, 2012). Again, ELSI scholarship emphasizes that technologies with biological or genomic components must be assessed in relation to the people, places, institutions, and social contexts in which they are deployed, rather than as isolated technical applications (Greenbaum, 2013; Trump et al., 2023).

Public and Indigenous engagement with oil sands remediation monitoring in Alberta has increased, particularly since 2017, when the Government of Alberta made changes intended to respond to criticisms about transparency and accessibility (Dubé et al., 2022). For example, Beckett et al. (2020) show that governments legally require public consultation for remediation, Beckett and Keeling (2019) discuss how a mining community sought support for community healing after remediation, and Blacker et al. (2021) highlight the growing use of citizen-science projects in post-remediation monitoring. However, meaningful collaboration with local and Indigenous communities remains limited in Alberta’s oil sands region (Aksamit et al., 2020). This limitation is significant because anticipatory governance and bottom-up ELSI approaches require the involvement of diverse actors before technical systems and rules are finalized (Matsuo et al., 2026). Existing industry collaboration has been organized largely around technological innovation and environmental performance, including COSIA’s (Canada’s Oil Sands Innovation Alliance) formation as an alliance among 13 competing oil sands companies and its later incorporation into Pathways Alliance (Slawinski et al., 2025; Aronczyk et al., 2024). Yet recent research on Pathways Alliance also shows that sector-wide environmental communication may involve selective disclosure, omission, and public-relations strategies that complicate trust in industry-led claims (Aronczyk et al., 2024). These concerns make transparency, accountability, participation, integrity, and capacity especially relevant governance principles for genomics-enhanced remediation (Trump et al., 2023). Since social science research in Alberta’s oil sands region remains “underdeveloped relative to other extractive regions” (Westman and Joly, 2019, p. 234), exploratory research on affected groups’ perspectives can help improve meaningful engagement and participation.

The study builds on Mont’Alverne et al.’s (2024) study of genomically-enhanced constructed wetlands researchers’ perspectives on their work and field, attending to concerns expressed by the researchers regarding the perceptions of genomics by different social groups (p. 6). For example, one researcher voiced concerns regarding how Indigenous communities may perceive microbes, “as [Indigenous Peoples] are perceived as having deeper respect for all elements of nature” (Mont’Alverne et al., 2024, p. 6). Here, we develop a comprehensive list of perspectives held by multiple groups of persons–including by Indigenous Peoples and of microbes–affected by oil sands remediation regarding the use of CTWS and genomics for OSPW, with the goal of identifying participants’ values and priorities for engagement with these aspects of remediation. These perspectives can help clarify the information by which decisions are made around oil sands remediation and, therefore, provide decision points for subsequent research and OSPW release criteria and regulations.

## 2 Materials and methods

### 2.1 Study design and context

This study was part of the 5-year, large-scale, mixed-method Genomics Research for Optimization of Constructed Treatment Wetlands for Water Remediation (GROW) project, situated in the component of the project that considers genomics and its environmental, economic, ethical, legal, and social (GE3LS) implications. A literature and media review was used prior to engaged methods to identify initial themes and opinion statements on oil sands remediation and genomics through the University of Saskatchewan USearch database, in Google searches for news and social media sources, as well as Twitter and Apple Podcasts. Social media channels are rich sources of ethnographical qualitative and quantitative data such as opinions and cultural trends (Giglietto et al., 2012; Snelson, 2016); thus, the search of popular social media and Google news sources was deemed relevant to the objectives of this work by supporting the cataloguing of existing perspectives for discussion. Literature search terms included “genomics,” “oil sands processed water,” and “constructed wetlands” as well as synonyms of those terms. Perspectives from these sources were drawn out as concrete statements for use in focus groups and one interview to initiate conversation, focusing on how they were written by experts or editors, or in public opinion.

Focus groups and one interview were used to understand the variety of perspectives around CTWS and genomics from four groups affected by oil sands remediation: Indigenous Peoples, oil and gas industry employees, CTWS and genomics researchers, and government regulators and policymakers considering OSPW remediation. Each focus group contained representatives from only one of these demographics, resulting in “homogeneity within the focus groups and heterogeneity between them” (Hay, 2016, p. 209). The shared characteristics of focus group members within groups supported the discussion of controversial topics, while the diversity between groups allowed for collecting a range of opinions (Hay, 2016). To ensure there were enough participants to encourage discussion but not so many that each participant did not have the opportunity to contribute, there were between five and seven participants per group (Hay, 2016).

### 2.2 Participant recruitment

Five categories of participants were recruited for focus groups. First, a pilot focus group was held as an essential step in tool design and development (Rattray and Jones, 2007) with a diverse group of faculty and student peers from engineering, environment and sustainability, and social science disciplines at the University of Saskatchewan. To recruit oil and gas industry participants for the second focus group, the lead author presented information about the research to the Canada’s Oil Sands Innovation Alliance (currently, Oil Sands Alliance) Mining Subcommittee. Following that presentation, attendees emailed lists of potential participants from their organizations. Similar recruitment processes were used for the third focus group, regulators and policymakers, through the Alberta Energy Regulator (AER) office and Alberta Environment and Protected Areas Agency (AEPA). Natural scientists were identified through Principal and Co-Investigators from the GROW project for the fourth focus group. One scientist participant could not attend a group session, so an interview was conducted using the same methods as for the focus group.

Finally, two of the other authors facilitated contact with potential Indigenous participants through their community-based and national and international research networks; these included Indigenous community members and scholars in the fifth focus group. This network-based purposive recruitment process then continued through snowball sampling, as initial contacts suggested additional potential participants. This approach is consistent with Indigenous-specific recruitment strategies that work through established relationships, personal networks, and participant referrals (Guillemin et al., 2016; Paine et al., 2013). See Table 2 for a summary of all focus groups and interview.

The five focus group sessions (Pilot, Industry, Regulator and Policymakers, Scientists, and Indigenous Peoples) and scientist interview included participants from Saskatchewan, Alberta, and New Zealand to understand the variety of perspectives around CTWS and genomics. The Indigenous Peoples focus group included persons from New Zealand due to their connections to Indigenous communities in the Canadian oil sands region through international research collaborations, for example, one Indigenous participant had resided and worked in Indigenous environmental and health research within the Canadian Prairies during the previous 5 year period. They were also included because of relevant Indigenous research relationships among colonized nations, and because of New Zealand’s legislative processes that were being implemented for developing regulations around the rapidly evolving fields of genomics and bioinformatics for remediation during the period. However, local Indigenous communities closest to the oil sands should be centered in any future decision-making.

### 2.3 Focus group format and materials

All focus groups and interview were held online and recorded through Zoom. Each session was approximately 60 min. The format included starting with introductions and describing the purpose of the focus group. The lead author used broad questions, such as “What comes to mind when you hear the term genomics in relation to remediation?”, and “What information would you need before supporting or opposing this approach?” and shared initial themes and opinion statements generated from the literature and media review (listed in Table 1) to prime thinking around the topic. More precise questions, such as “What benefits, risks, or concerns should be considered before CTWS enhanced by genomics are used?” were asked to elicit specifics from participants (Nyumba et al., 2018; Stewart and Shamdasani, 2014). Padlet (http://padlet.com/), an emerging tool for educational and research settings, was used as a virtual workspace and visual tool to display and develop themes and opinion statements with the focus groups (Deni and Zainal, 2018). This approach also parallels interactive ELSI methods such as risk mapping, which use structured discussion to expand the range of risks and relationships considered in governance (Akyüz et al., 2024). The pilot recording was transcribed manually by the lead author; the remaining recordings were transcribed by Rev.com and then reviewed manually for accuracy by two members of the authorship team. Transcripts were coded in a thematic analysis, and this is what we discuss here. Additional themes and opinion statements were developed with the focus groups and interview for use in Q-methodology and are discussed elsewhere (Tilford-Shaw, 2023).

**TABLE 1:** Collated initial opinion statements sourced from literature and media review.

*Collated initial opinion statements #*	Initial opinion statements (literature and media review)	Initial themes (literature and media review
1	I associate genomics with genetic modification	Confusing
2	Because I do not understand how genomic study results are presented and ultimately used, I do not want genomics used for oil sands process-affected water remediation	Confusing
3	I like constructed wetland treatment systems to treat oil sands process-affected water because they are cost-effective	Innovative
4	I like constructed wetland treatment systems to treat oil sands process-affected water because they are easy to operate	Innovative
5	Genomics holds the key to developing innovative biological strategies, allowing us to work towards a more environmentally friendly approach to monitoring, managing, and treating mining wastewater	Innovative
6	I like constructed wetland treatment systems to treat oil sands process-affected water because they are natural	Natural
7	I support anything that can improve the processes of environmental cleanup	Natural
8	Anything that can mimic nature has a better chance of success	Natural
9	Too much technology causes problems	Natural
10	Genomically-enhanced constructed wetland treatment systems sound like the best option out of anything anybody’s come up with so far	Resignation
11	Bioremediation brings uncertainty, but some types of uncertainty are reducible in principle if the requisite knowledge can be produced	Resignation
12	I support genomically-enhanced bioremediation if it is coupled with monitoring	Resignation
13	I worry that microbes could escape from the constructed wetland treatment system and disrupt or displace native microbes, upsetting ecological balance and potentially knocking something else out that’s really critical in the natural environment	Unknown impacts - microbes
14	While the microbes may be able to sequester toxic materials, I worry that these do not disappear but remain in the sediment, from where they could work their way into natural systems and become available to food webs	Unknown impacts - microbes
15	I need more information about the microbes and side effects to the environment and people from microbial remediation before I will support it	Unknown impacts - microbes
16	I think toxins will be released from these systems and worry about the potential cumulative impacts of these “new” contaminants in combination with existing sources of pollution in the watershed	Unknown impacts - Unplanned release
17	I worry natural accidents such as floods, storms, droughts, and earthquakes could destroy the constructed wetland treatment system and release sequestered toxins and microbes into water systems	Unknown impacts - Unplanned release
18	I worry small accidents, leaks, and so on may go unnoticed until the system collapses	Unknown impacts - Unplanned release
19	The knowledge necessary to reduce uncertainty for constructed wetland treatment systems cannot be produced	Unpredictable
20	Not only do ecosystems shift and evolve over time, but issues such as climate change affect our ability to predict future conditions	Unpredictable
21	I do not like the biological complexity of constructed wetland treatment systems as it makes predicting impacts difficult	Unpredictable
22	I support inoculation of wetland plants with microorganisms having specific metabolic capacities and/or plant growth promoting activities	Unpredictable

**TABLE 2:** Focus group summary.

Participant category	Number of participants	Focus group type (focus group or interview)
Pilot (P)	5	Focus group
Oil and gas industry (I)	7	Focus group
Regulatory and policymaker (R&P)	6	Focus group
Scientists (S1)	1	Interview
Scientists (S2-5)	5	Focus group
Indigenous peoples (IP)	5	Focus group

### 2.4 Focus group thematic analysis

Thematic analysis of the focus group and interview transcripts was completed by the lead author through open-coding line-by-line (i.e., including wording that participants used in the focus groups and interview; Glaser, 2016) in NVivo. Initial themes from open coding were shared with authorship team members for discussion of relevance to previous literature and novelty. The second coding cycle included having codes collapsed into axial codes or themes by three authors through in-person discussion to enhance triangulation (i.e., as per Santos et al., 2020) to confirm similarities and discrete participant perspectives of CTWS and genomics.

## 3 Results

**FIGURE 1:**
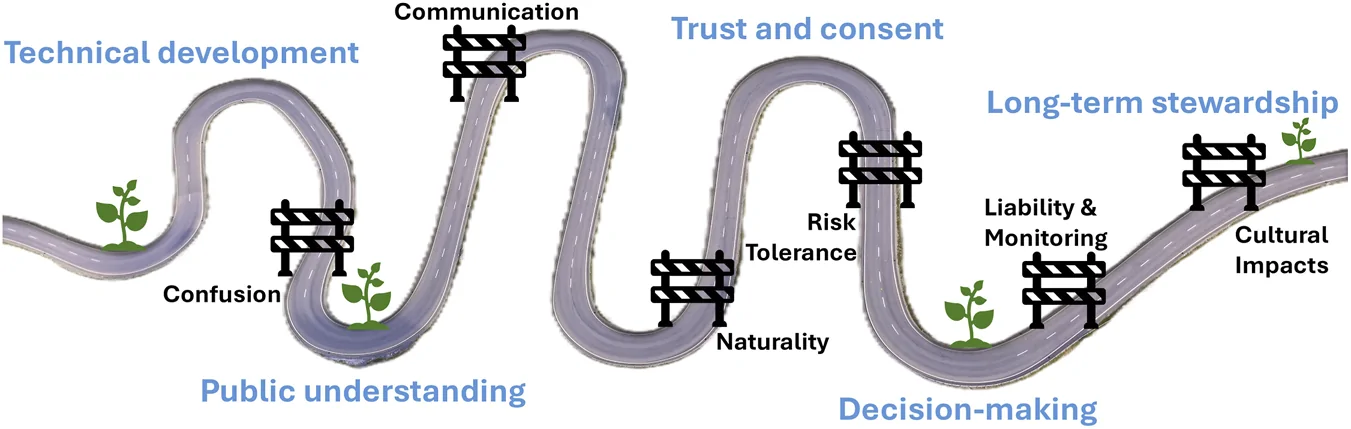
Six barriers to participation in oil sands remediation using genomics-enhanced constructed treatment wetland systems.

Thematic analysis of focus group and interview transcripts identified six main themes in participant perspectives that underlie barriers to participation in oil sands remediation with genomics-enhanced CTWS: (1) confusion; (2) communication; (3) naturality; (4) risks; (5) liability and monitoring; and (6) cultural impacts (see Figure 1). These have resonance with the initial opinion statements and themes identified in the literature and media review. In the evidence presented next, members of focus groups and interview are identified by the type of participant (i.e., P = Pilot, R&P = Regulator and Policymaker, I = Industry, IP = Indigenous Peoples, S = Scientists).

### 3.1 Theme 1: confusion

Participants expressed confusion about a variety of terminology and concepts involved with CTWS and genomics, including what they are and how they intersect. Most participants had some prior knowledge of CTWS, though they acknowledged that people without a bioremediation background may find the wetland systems confusing, and some admitted to being unsure of what the wetland systems involved. Most participants also admitted being confused about genomics, and many specified having concerns about microbes. For example, many participants said that language around genomics is unclear, as stated by an Indigenous participant:

There’s a lot of confusion about the terminology of genomics. As I’m reading it, it just looks more like a reconstruction of the ecology. Am I correct? You guys are not doing any kind of CRISPR-type activities on the microbes or the plant material that you’re looking in there. You’re using the genomics part essentially is like eDNA, you’re figuring out what species would exist in a natural wetland and then trying to reconstruct it in a space that where you need to treat the industry that’s extracting oil sands. Is that correct? (IP4)

One participant from the regulator and policymaker group explained that even those with a technical background in CTWS and genomics can become confused about the concepts:

When you talk about genomics, I’m thinking about genetic modifications in terms of living organisms, whether that’s plants or animals. And so, in this case, if we are talking about constructed wetlands, we’re talking about processed water, I cannot immediately make a connection between the term genomics and how the processed water which is mostly the chemical constituents of that would be related then to constructed wetlands. (R&P3)

Some participants acknowledged that although they did not have an effective understanding of genomics, they might support its use if they did. One participant from industry put it this way: “I’m not opposed to genomics being used for water remediation. I just need to be more informed of what it is… If it works, go ahead and use it” (I4). Another, from the regulator and policymaker group, echoed that sentiment: “I’m not opposed to it or anything like that. I just need more information to understand it better” (R&P2). An Indigenous participant said that they took “a cynical and very skeptical position” about genomics due to their uncertainty about the end use for the data generated: “What is it actually for and who actually benefits? Because depending on how that data is managed and controlled, the ability of systems now to just store data until they find some use for it is phenomenal” (IP5).

While there was confusion about CTWS and genomics, many participants said that discussions like those in the focus groups helped identify learning opportunities. Participants taught researchers and each other new things by correcting or challenging statements from the literature and media review and earlier focus groups and/or interview. Other participants acknowledged that identifying perspectives, even confused ones, provides the space to learn. As one Indigenous participant put it,

Just to be able to have those conversations with community members, I think it’s really important. And a project like this would bring that opportunity for us to have these types of conversations and educate ourselves. So, I do think there’s an education piece here, even good or bad, I think all of those pieces are important to understand. (IP2)

This opinion was shared by a participant in the scientist focus group:

I do not know much about this kind of form of research, but I wondered if gathering these types of opinions creates an opportunity for “What do we need to educate people about … ?” It can serve as like, “Okay, this is our jumping off point for education,” right? There’s a lot of misconceptions… We can, scientifically, contribute to the proper definition, and context. (S3)

Participants of this study generally agreed that discussions about CTWS and genomics with diverse groups of people affected by oil sands remediation create value through identifying knowledge gaps and opportunities for these people to teach and learn from each other.

### 3.2 Theme 2: communication

In part to address the confusion described in the prior section, many focus group participants and the scientist in the interview also stressed the importance of plain language, clear definitions, and context used to communicate about CTWS and genomics. One Indigenous participant (IP3) drew from their experience with impact assessments to recommend including explanations in plain language. Another Indigenous participant (IP4) suggested developing a common language dictionary. While a participant from the regulatory and policymaker group (R&P1) put the onus on industry to ensure that CTWS are clearly explained to all stakeholders, others indicated that responsibility should lie with researchers as well. Some participants recommended that researchers should make a clearer distinction between natural and constructed wetlands, such as this participant from the regulatory and policymaker group, who suggested, “Somewhere you have to clearly say, are we using the constructed or engineered or treatment system, or we are using the natural one that is available in the ground and just we discharge the wastewater?” (R&P4). Many participants indicated that researchers and industry should present the background and details of CTWS and genomics more clearly to remove as much ambiguity as possible. Many also suggested that providing more context about microbes could eliminate the confusion around them, as well.

Participants agreed that the way that information is presented can influence opinions, and that great care must be taken when selecting language and framing communications about research and regulatory discussions. For example, one Indigenous participant voiced concern over how information and research is presented to Indigenous communities:

Just listening to some of the comments, I do think about issues like framing bias and how you frame things will direct people on how they respond. And I think if you are just saying as a general principle, cleaning up the environment is a good thing. Yeah, you’ll get everyone from our communities to agree, but I do not think that that logically means then that therefore we must support this project because this is the best way to do it. (IP3)

The same Indigenous participant (IP3) also discussed the importance of not only communicating information to Indigenous communities but also for industry and government to really listen to them. Specifically, this participant emphasized the importance of involving the communities who are closest to the proposed remediation:

It’s not something that should be taken lightly and really needs to be done in collaboration with the leadership and local communities up there and make sure that they’re comfortable with this. Because I appreciate that I am from Treaty Eight but I’m not from that region of Treaty Eight and I would defer to them and their concerns, but certainly making sure that they’re a key part of this process and can help make decisions, and that you’re thinking about how do you address the concerns that they’re going to raise. (IP3)

### 3.3 Theme 3: naturality

Participants shared conflicting viewpoints whether CTWS are natural interventions. For example, one industry participant described CTWS “as a way to learn from nature because there are already opportunistic natural wetlands that are doing treatment by itself. And we’re basically the wetland treatments are that is just taking that, I guess knowledge and wisdom from nature and applied that for treatment” (I7). Another industry participant questioned if CTWS would be less natural if building the systems “disturbed a lot of natural area,” adding, “I’m wondering if there’s something to be said about the size of these wetlands that would make them maybe not as natural if we need a big, a lot of hectares to basically process this water” (I6).

Participants from the regulatory and policymaker group were less inclined to view CTWS as natural. One participant distinguished CTWS “as using biological processes, but I do not look at them as being natural” (R&P6) and another as engineered systems in contrast to natural ones: “I’m talking always engineered constructed wetland that is designed and it is a treatment system. Let’s not talk about the natural, I’m not talking about natural treatments, natural wetland” (R&P4). Yet another participant from the regulatory and policy making group questioned whether the naturality of CTWS is important or relevant to remediation processes:

If you can scroll up just to, where was it? Oh yeah, “anything that can mimic nature has a better chance of success,” and as well as kind of, I do not necessarily agree with that. I think… that there’s a lot of kind of uncertainties. Knowing things that wetlands are not able to treat for as well. So yeah, making that assumption that if it’s a natural or if it mimics nature that it’s going to be the solution is again quite naïve. (R&P2)

An Indigenous participant also problematized the question of naturality, though in a different way. This participant posited that the Western view of “nature” itself may differ from Indigenous perspectives:

Different worldviews can be applied to every category, even the use of categories. So now we have the term “natural” and “nature”, and this is one of the classic dichotomies from colonial viewpoints would be there is the natural world and there is the human world and they’re different things… And [IP4] mentioned it earlier, the familial relationships that Indigenous peoples have with their environment, relationships as family… And so, to construct nature as a different system is a worldview. (IP5)

The apparent complexity within the question of naturality suggests that there are multiple different perceptions of these remediation options that underlie these groups’ engagement with them, some of which may be due to different cultural backgrounds. Indigenous perspectives of wetlands as kin may yield very different considerations for risks than may come from a Western perspective. For example, while this Indigenous participant’s questions similarly consider naturality, another also considered the wetland’s identity:

For me it would be a case of are we putting an ecosystem into a space where it did not naturally exist before, or are we recreating the ecosystem that used to be there? Is that the song that that landscape used to sing, or are we forcing it to sing off another song sheet? (IP4).

### 3.4 Theme 4: risk tolerance

Like the responses to the question of CTWS naturality above, differences in risk tolerance regarding CTWS and genomics also appear to be affected by participants’ differing backgrounds. While all participants agreed that CTWS enhanced by genomics face substantial amounts of unpredictability, such as that which is related to the potential effects of climate change on the local conditions in which pilot CTWS’s have been studied, only some felt that these risks associated with CTWS and genomics are mitigated or justified. Participants expressed that the pilot systems implemented in the oil sands are adequate to prove the efficacy and safety of a treatment method. One industry participant (I2) thought that sharing the multiple other wastewater cleanup applications of CTWS may help alleviate some of the discomfort and negativity that affected persons may feel about CTWS approaches. Another industry participant raised a similar argument in favour of genomics, e.g., that it is “used in other industries” (I4). However, an Indigenous participant disagreed with that viewpoint: “Success in an industry essentially means it has contributed to growth in that industry, which does not mean a success for wider society, let alone Indigenous communities in there” (IP5). Another Indigenous participant built on the previous comment, adding, “I think that for me, I would want to see some proof that there would not be impacts, significant impacts to Indigenous communities there before” (IP3). The same participant (IP3) concluded that, “until you can prove no harm will result from doing this, you should not do it.”

Other participants commented on the magnitude of OSPW, with one participant from industry commenting, “I like constructed wetland treatment systems because they’re scalable… probably that one of the few treatment water treatment options that scales, you know, when you scale it up, it only works better” (I3). This viewpoint was not shared by all participants, such as this scientist with a background in remediation: “Well, oil sands process-affected water… it is not that the water is wildly contaminated. I mean, there’s a lot of other really nasty wastewater streams that are much more difficult to deal with. It’s the scale that’s really the challenge in treatment” (S1). Others–here, one from the regulatory and policymaker group–raised concerns about reclamation complications with large CTWS:

From a reclamation and closure perspective, a thought has to be put into the acceptance or what these treatment constructed wetlands are going to end up in the closure landscape and if that’s acceptable, which has already been said. So scalable is one way to put it, but you really do not want to have too many of them or larger extents of them on the landscape, because we do not know as yet if there is possible or how expensive it would be then to get that constructed wetland back to let’s say a natural wetland or even a lake after everything is gone. So, I think the closure aspect has to be thought of. And yes, it’s been mentioned, but there’s unknowns. And so, the idea of making this scalable and getting them larger and larger may not be acceptable from a stakeholder perspective. (R&P5)

Some participants questioned the ability of CTWS to handle the many contaminants present in OSPW as a standalone remediation process. Indeed, many, such as this participant from the scientist group, emphasized that CTWS enhanced by genomics should *not* be used as standalone methods and would need additional steps before and after their application to wetlands to ensure the water meets stringent release criteria:

I’m in the camp of there is not one treatment method for the water. That’s not saying that we need a train of super highly engineered treatment systems all working in tandem. But I think you’ll need other forms of treatment, and they could be very passive forms, but there there’ll be other needs for the water. It’s not a, “This will go from the holding pond through the wetland straight into the river.” It’s going to require either, at its simplest, and I know people do not view this as treatment, but different mixing strategies on site. Because it’s not going to remove the salinity of the water, which is what will be a more contentious issue amongst different groups that are managing. (S1)

This participant from the regulatory and policymaker group also explained why additional treatment will be required:

If I look at the oil sands mines, the chemicals present, they are toxic. Let’s say very difficult to synthesis by bacteria. So the treatment systems are good for bacteria that can consume biodegradable materials like BODs. But there are some chemicals which cannot be removed by treatment systems. One example I’ll give you is PAHs, poly-aromatic hydrocarbons. Another solid example is selenium. Removal of selenium is a problem. And you have to create anaerobic condition as well as aerobic condition. And I have not seen any treatment technologies using constructed wetland. So, there are some limited uses of treatment systems using the constructed wetland. (R&P4)

In short, multiple participants agreed with the view that genomics and CTWS present one of a suite of options, each with “known unknowns” and “unknown unknowns,” rather than a single transformative solution. Together, these differing views on the risks involved with CTWS and genomics point to the importance of considering the implications of cultural and social backgrounds in making decisions about remediation.

### 3.5 Theme 5: liability and monitoring

Questions about the long-term effects of CTWS enhanced by genomics highlighted concerns about who would hold liability for, and continue the monitoring of, these CTWS. Regulatory and policymaker participants stressed that there must be a “distinction [drawn between] using constructed wetlands on… a closure landscape [the after-mining landscape], versus while things are still in operation” (R&P6), and that, “for the area to be considered permanently reclaimed, they cannot have an operational constructed treatment system of any sort, whether it is a wetland or a more engineered kind of system” (R&P2).

One scientist suggested that wetlands will be an inevitable feature of the landscape after the oil sands mines are closed:

I think this, maybe, comes back to one of the phenomena that we’ve come across in our work, insofar as, in that region, in that muskeg heavy sort of landscape, where you have considerable dry years, but considerable wet years, whether or not, you, intentionally, place wetlands on the closure landscape, there will be wetlands on the closure landscape, through settling, and compaction, and water finding its path to the low points. They’re going to be a part of the landscape, one way or another, so I think that adapting to their presence, whether constructed, opportunistic or otherwise, is probably still wise and relevant (S5).

Indeed, another scientist saw the ability to become a permanent part of the landscape as an advantage of CTWS enhanced by genomics:

I see that as a benefit of this system where you can transition it to a state that could serve a lot of ecological services and be a good feature on the landscape potentially in addition to providing treatment during the more active phase. So, I’ve always viewed that as a benefit. (S1)

Industry participants argued that liability is held by oil and gas companies and that adaptive managing and monitoring would be part of their plan. One participant elaborated:

Once we build whatever our closure profile is going to be, whether it’s a wetland treatment, whatever, we’re not going to be able to walk away from these facilities until we’ve proven that these things are stable, reliable and work. It’s part of our due diligence is that we’re not just going to be able to say, okay, the wetland’s built, yeah, okay, sign off on it, we’re all done. That’s not going to happen. We’re going to have to do years and years and years of monitoring and so on to make sure that these work … And if they do not work or there’s a problem, then we’re going to have to do some mitigation to make sure that they work or to fix it. So, I do not know how to put that in, but it’s not as if we’re going to be able to build this and walk away. We’re going to have to build it, monitor it, maybe have to do some adaptive management during the process, but we’re, we’re going to have to monitor and maintain these for likely decades after they’re built to ensure that they do what they say they’re going to do, because there’s not a lot of trust. (I4)

An Indigenous participant was concerned about how the Canadian government has dealt with liability for negative environmental impacts in the past:

The response from the Crown and the response from proponents [following consultation with Indigenous communities] is always, “Don’t worry, we’ll take care of all the problems.” And then suddenly, it’s as if you wave the magic wand and all the problems that the communities are concerned about have gone away. And really that’s not true. There is almost nowhere left in Northern Alberta that you might have called a virgin territory. In my grandfather’s time, you could go to any body of water and drink the water. You could harvest any plant or any animal and eat it safely without concerns about toxicity or bioaccumulation of toxins. And so, that’s part of the challenge here…. By and large, there’s not a lot left there that has not been touched or scarred by development. (IP3)

This theme emphasizes that both unknown futures and known histories pertaining to liability and monitoring must be addressed before OSPW remediation methods may be accepted by affected persons.

### 3.6 Theme 6: cultural impacts

Participants in all focus groups and interview stressed the importance of considering cultural impacts. Many participants tried to imagine themselves in the position of community members, expressing sympathy and offering statements like this pilot participant: “If I was living close by, I would not want to see a forest come down to fit in a, a wetland that someone’s constructing” (P2).

Discussions of burden and cultural impacts in all focus groups and the interview brought forth the need for collaboration with local communities and Indigenous decision-making. This was especially salient for the Indigenous participant group. For example, one Indigenous participant said that heightened Indigenous standards regarding the precautionary principle highlighted the need for Canadian and American governments’ collaboration with Indigenous communities:

What I’ve noticed is that the Canadian government, the Alberta government, the American government standard is usually on the very, very low end. Go ahead and do it and then if something bad happens, we’ll respond and react later. Whereas Indigenous Peoples tend to take a much stronger point of view as it relates to doing these sorts of things. (IP3)

The same participant spoke about the United Nations Declaration on the Rights of Indigenous Peoples (UNDRIP) Act, Free Prior and Informed Consent (FPIC), and the duty to consult. Another Indigenous participant asked, “Is it then okay for me to suggest a statement that says that Indigenous peoples have the right to veto at any stage of that, that they do not feel comfortable, and the precautionary principle is not being addressed?” (IP4). These comments suggest that participants were not only asking whether CTWS enhanced by genomics could work technically, but also who should have authority to decide whether the risks are acceptable. This question of authority led directly into concerns about responsibility, care, and the uneven burden placed on Indigenous Peoples when remediation interventions alter living landscapes, discussed in the previous section.

One concern, related to the different ways that the environment is perceived from within Indigenous worldviews, was on where the burden of remediation is placed. An Indigenous participant, for example, thought that CTWS enhanced by genomics could place a greater burden on Indigenous Peoples due to their cultural values regarding responsibility and care for the Earth:

There’s always a concern that if you are recreating a system in a place that it has not been before, not only are you recreating a system on the land that it does not recognize it, but you could also be inadvertently introducing new things into those spaces even though they’re native or they’re endemic to the region. And that can sometimes put quite a lot of pressure on the Indigenous People at place who may then feel compelled spiritually and culturally that they have to take responsibility for the care of those things as the people of that space. And so I guess the thing that concerns me across the whole thing is bearing in mind it might be native, but you could be introducing it to a space where it never was seen before or felt or tasted before, and the pressure that it then puts on that local Indigenous community to then become the guardians or the protectors of those things that are not from their space naturally (IP4).

In this framing, the cultural impact is not limited to whether CTWS are physically safe or technically effective; it also includes whether remediation shifts ongoing responsibilities for care, protection, and relational accountability onto Indigenous Peoples. The above and other potential cultural impacts shared by focus group participants are perhaps made more critical by the perspective, held by some participants and related to the view of wetlands as kin, that wetlands have a spirit–and that CTWS may not. One non-Indigenous pilot participant expressed their understanding of Indigenous worldviews in this way:

Everything, everything has a spirit. Um, like this rock is, has spirit to it, but as soon as humans take and manufacture something, it loses that- it not necessarily loses it, but it does not have it. Anything human made does not have that spirit. (P3)

Other participants (e.g., scientist participant S3) who did not share this belief expressed respect for it. The perspectives of wetlands as kin and wetlands with spirit may have consequences when making decisions about CTWS enhanced by genomics, including determining the burden of remediation and how remediation is done. These participant thoughts and experiences make the case for collaboration with local communities–and especially with Indigenous communities–to address concerns with the cultural impacts of CTWS enhanced by genomics for OSPW remediation.

## 4 Discussion

The findings of this research provide a bearing for ELSI researchers because OSPW remediation supported by genomics is not a distant or abstract technical problem. Tailings ponds already occupy a large area of northern Alberta, continue to grow, and remain tied to closure assumptions that include eventual treatment and release of remediated water (Environmental Defence Canada and Canadian Parks and Wilderness Society, 2022; Quinlan and Tam, 2015; Slingerland et al., 2019; Tanna et al., 2019). In this context, decisions about CTWS enhanced by genomics will shape not only contaminant reduction strategies, but also long-term responsibilities for monitoring, liability, communication, Indigenous rights, and cultural continuity. This aligns with ELSI scholarship arguing that genomic and biotechnology applications cannot be assessed only through technical performance; developers must also address safety, security, transparency, social acceptance, and the broader ethical, legal, and social conditions under which technologies are produced, deployed, and governed (Greenbaum, 2013; Trump et al., 2023).

The main barriers to participation in OSPW remediation using CTWS enhanced by genomics identified in this study were a lack of understanding, particularly about genomics and how CTWS and genomics intersect; ineffective communication, with a need for more apparent context and more accessible terminology; and lack of awareness of different viewpoints. The reasons affected persons gave for personally supporting CTWS enhanced by genomics were trust in pilot projects, reassurance that both CTWS and genomics are being used successfully elsewhere, an understanding that research has been done to study the methods, and that research will be ongoing (Alvarez Aguilar, 2024; Alvarez Aguilar et al., 2026) submitted for publication; Tilford-Shaw, 2023). Participants emphasized that CTWS are not a standalone method, and this study found a range of views about the acceptability of CTWS on a closure landscape. Some participants thought that a benefit of CTWS is the ability to naturalize and provide ecosystem services. Others were adamant that CTWS would have to be removed for the land to be considered reclaimed, since CTWS are limited in what they can treat, and because of regulatory requirements for closure profiles. The main arguments against CTWS enhanced by genomics were that there is not enough information and there is no proof that this treatment method will not cause harm to the environment, wildlife, or human health and culture–especially considering Indigenous Peoples’ proximity to closure landscapes, and their livelihoods and cultural continuity. These perspectives affirm findings by Page and Atkinson-Grosjean (2013).

Figure 1 Synthesizes the six ELSI barriers identified through the focus group and interview analysis as obstructions on the pathway toward meaningful engagement with CTWS enhanced by genomics for OSPW bioremediation. The road metaphor also reinforces Page and Atkinson-Grosjean’s (2013) caution in that that positive attitudes toward ecogenomics-enhanced remediation should not be interpreted as final acceptance of a technology. In arranging the barriers as a sequence of obstructions, the figure also extends previous findings by showing a prioritized pathway for improving public perspectives and possible uptake: confusion and communication must be addressed before deeper concerns about naturality, risk, liability, monitoring, and cultural impacts can be meaningfully discussed. Figure 1 frames engagement not as a single consultation step, but as a staged process of building shared understanding, trust, and decision-making capacity.

This study also extends Mont’Alverne et al.’s (2024) work on genomics researchers’ perspectives by showing that researchers’ concerns about public, Indigenous, and cross-sector perceptions of genomics were well-founded, but incomplete. Participants’ responses suggest that the challenge is not simply public misunderstanding of genomics or microbes. Rather, acceptance of CTWS enhanced by genomics is influenced by governance, authority, trust, communication, liability, cultural responsibility, and the perceived adequacy of proof–hallmarks of free, prior, and informed consent.

### 4.1 Barriers from confusion

Participants confirmed Page and Atkinson-Grosjean’s findings (2013) that genomics is polarizing and misunderstood, especially when considered with CTWS. The repeated themes of confusion and uncertainty within the focus groups and interview reinforced the statements and themes of the literature and media reviewed; genomics research is advancing at a rate at which public education about genomics technologies cannot keep up. This knowledge gap creates challenges around public support of genomics-based methods to enhance remediation. However, our findings suggest that confusion should not be treated as a simple absence of information or lack of westernized education. Participants asked whether genomics meant genetic modification, environmental DNA analysis, microbial monitoring, or a form of ecological reconstruction, showing that the participants are knowledgeable and informed, but the term carries different meanings across technical, regulatory, and cultural contexts. This finding reflects broader ELSI concerns that public trust in biotechnology can be undermined when scientific terminology is unclear, when past controversies frame current interpretations, or when the purposes and boundaries of the technology are not transparent (Greenbaum, 2013; Trump et al., 2023).

### 4.2 Barriers from communication

To implement CTWS enhanced by genomics, areas of confusion among affected persons must be addressed with meaningful communication and engagement. Such communication and engagement have the potential to develop the trust required to work together in addressing the wicked problem of OSPW remediation: trust in its methods, communication, and language, in the people in power, among actors and viewpoints, and in the monitoring. The great debate about whether CTWS are natural or not highlights the importance of the language used in oil sands remediation communications. This finding is particularly important in a sector where environmental communication is already politically charged. Participants’ concerns about framing bias, plain language, and who is responsible for explanation resonate with recent critiques of sector-wide environmental communication, including concerns about selective disclosure, omission, and public-relations strategies in oil sands climate messaging (Aronczyk et al., 2024). In this context, communication about CTWS enhanced by genomics must do more than promote potential benefits; it must make uncertainties, limits, responsibilities, and decision-making authority visible.

The findings also align with ELSI risk-governance scholarship emphasizing that trust and sustainability are cross-cutting elements of risk assessment and that ethical governance requires expanding the range of risks considered beyond technical failure alone (Akyüz et al., 2024). For this study, such expansion means treating confusion, terminology, relational trust, cultural burden, and monitoring responsibility as part of the risk landscape rather than as secondary communication issues.

All focus groups and the interview acknowledged the need for meaningful collaboration with local communities (Aksamit et al., 2020), and the study confirmed concerns in the literature about a lack of engagement and communication about OSPW remediation measures, resulting in an absence of trust between affected persons (Aksamit et al., 2020). Participants in this study echoed Westman and Joly’s findings (2019) that the individuals and communities most impacted by OSPW remediation feel marginalized, powerless, and under-informed, referencing issues with policy, including UNDRIP, FPIC, and the duty to consult.

### 4.3 Barriers from different perspectives

This study reinforces earlier findings (Page and Atkinson-Grosjean, 2013) regarding the importance of learning whether local communities will accept remediation technologies and what factors influence their level of acceptance. People consider many factors before deciding to support a remediation method. One factor that deserves greater attention is the relationship between uncertainty, climate, and proof. Because northern Alberta’s harsh climate and changing seasonal conditions may affect CTWS performance in ways that are difficult to replicate fully in controlled settings, participants’ requests for more information should not be read as resistance to innovation, but as a call for evidence that is locally meaningful and temporally durable (McQueen et al., 2017; Trepanier et al., 2025). The finding aligns with anticipatory governance scholarship, which argues that emerging technologies require proactive attention to future social and environmental impacts before governance systems fall behind technological development (Matsuo et al., 2026).

A further important factor includes affected peoples’ perspectives on the naturality of CTWS and genomics, the risks involved, monitoring and liability, and the nature of wetlands and the Earth themselves. Some of the latter perspectives, particularly the beliefs that wetlands have a spirit and may be understood as kin, were new to many participants outside the Indigenous Peoples group. These perspectives invite dialogue beyond technical risk assessment by asking how remediation decisions should account for relationships, responsibilities, and forms of life that may not be captured through conventional regulatory categories. From an ELSI perspective, this uncertainty also makes anticipatory governance important because oversight, engagement, and rulemaking can lag behind technical development (Matsuo et al., 2026).

Although the literature has identified CTWS as one of the few technologies with the potential to handle the magnitude of OSPW and its complex mixtures of contaminants, the many different perspectives identified may affect participants’ participation in oil sands remediation. For example, some participants liked the scalable nature of CTWS to treat the large volume of OSPW, but others did not want large treatment systems on the landscape. Findings such as these suggest that more research is required to support local decision-making on acceptable remediation plans. The wide diversity of viewpoints suggests that it would be challenging to pinpoint exactly what community improvement looks like in practice, thus highlighting the importance of understanding the opinions of local community members who are most affected.

### 4.4 Liability, monitoring, culture, and closure as ELSI issues

Questions about liability and monitoring make the ELSI dimensions of CTWS enhanced by genomics especially concrete. Participants did not ask only whether the systems could function; they asked how long monitoring would continue, who would remain responsible if performance changed, whether CTWS could be part of a closure landscape, and whether an operational treatment system is compatible with permanent reclamation. These concerns connect technical uncertainty to legal and institutional accountability, especially because OSPW remediation and release are tied to mine closure assumptions and long-term environmental governance (Tanna et al., 2019).

The research findings support ELSI governance work arguing that risk assessment should be conducted early, repeatedly, and transparently, and that biotechnology governance must include accountability, participation, integrity, and capacity rather than focusing only on technical risk (Trump et al., 2023). It also echoes Akyüz et al.’s (2024) argument that risk governance requires identifying, assessing, mitigating, and communicating risks while expanding the repertoire of risks considered. For CTWS enhanced by genomics, that expanded repertoire includes liability after closure, adaptive monitoring, responsibilities for failure, the credibility of monitoring institutions, and the ability of affected communities to access and interpret monitoring data.

The findings on cultural impacts also show that cultural assessment cannot be reduced to physical footprint, toxicity, or technical performance. Participants’ comments about wetlands as kin, wetlands with spirit, and Indigenous responsibilities for care suggest that remediation can alter relationships, duties, and obligations, even when the biological materials used are native or locally occurring. This finding extends Westman and Joly’s (2019) discussion of subsistence, spiritual practice, and intergenerational knowledge transfer by showing that remediation itself can create cultural impacts if it introduces new responsibilities for guardianship or protection.

These findings also connect to Indigenous research ethics literature. Guillemin et al. (2016) show that Indigenous participants may approach research from a position of caution, assessing honesty, reciprocity, respect, and community benefit rather than taking researchers’ claims at face value. In this study, similar forms of caution appeared not only toward the research process, but toward remediation decision-making itself: participants asked who benefits, who decides, who has veto power, and whether Indigenous Peoples would be left to care for altered landscapes.

### 4.5 Limitations of methods and materials

As with all focus groups, there is the potential for cohort effects due to geographical, temporal, or other global or local events to influence the sample’s perspectives, observation and social desirability biases, and language constraints with participants who use English as an additional language. Members of the focus groups expressed opinions in line with the roles they hold, but it can be questioned how much the homogenous focus group setting influenced their responses: were participants saying what was expected of them as oil and gas company employees or regulators, or would their responses have been different using a different method like the individual interview?

While most of the focus group conversations were around CTWS and genomics and their potential effectiveness and impacts, some participants also discussed researcher methods. While some participants responded well to the online method and Padlet, others expressed concerns regarding the technology used. The online format made connection more difficult, so there was less rapport between participants than necessary to call this truly successful transdisciplinary research. Although the Padlet initially worked well, its effectiveness decreased as the number of statements increased. Additionally, the Padlet was not well received by some groups; some participants did not feel that the approach felt inclusiveit and others strongly disliked the approach. One participant said, “It’s really hard I think as an Indigenous person to break it down into these columns like this. It’s a lot easier sometimes for us to be able to go boof and go over the whole thing” (IP4). Participants often challenged the themes and statement placement, with participants saying things like, “I have another statement that I do not know where to put it. Maybe might be in the natural part, but to me, it is the fact that maybe it is also related to spiritual, I do not know” (I7).

While the lead author adjusted to feedback that the presentation of information was not conducive to discussion with participants in the Indigenous Peoples session, it was difficult to modify methods mid-session and scheduling restraints did not allow for follow-ups. These methodological questions align with the complexity discovered in the thematic analysis: there are wide-ranging viewpoints on using genomically-enhanced CTWS, as well as on how to study the human dimensions of these systems.

Thus, the methods themselves became part of the findings. Although Padlet and the online format helped structure discussion, display statements, and allow participants to compare perspectives, some participants found the column-based format constraining, especially when their comments crossed categories such as natural, spiritual, cultural, and risk-related concerns. This methodological tension matters because ELSI research seeks to move beyond collecting views on emerging technologies; it is also about designing engagement formats that do not prematurely sort, narrow, or translate participants’ ways of knowing into researcher-defined categories. The findings therefore support ELSI scholarship calling for bottom-up deliberation and more proactive involvement of research communities in governance, while also showing that bottom-up methods must be flexible enough to respond to participants’ own epistemic and cultural frameworks (Matsuo et al., 2026). They also align with risk-mapping approaches that use interactive discussion to expand the set of risks recognized by technical and governance actors (Akyüz et al., 2024).

## 5 Recommendations and conclusion

The overall goal of this study was to gather insights into perspectives about OSPW remediation using CTWS enhanced by genomics. The study found that while some persons affected by oil sands remediation have strong opinions about the treatment method, many are unsure or confused about it and want more information. Such a gap presents a barrier to participation in oil sands remediation. The study highlights the information that people use to make decisions and therefore supports subsequent research and regulation processes.

The study identifies several decision points that should be addressed in future research, regulation, and engagement about CTWS enhanced by genomics. These include: how genomics is defined and distinguished from genetic modification; whether CTWS are presented as natural, engineered, or hybrid systems; what evidence is sufficient to demonstrate safety and efficacy under northern Alberta conditions; how treatment trains will address contaminants that CTWS alone may not remove; who will monitor performance over time; who will hold liability after closure; how Indigenous authority, FPIC, and veto concerns will be handled; and how cultural and spiritual impacts to wetlands and surrounding landscapes will be assessed. These decision points are consistent with ELSI and safety-by-design approaches that call for risk, governance, and social implications to be considered early and repeatedly rather than after a technology is already near implementation (Trump et al., 2023). They also reflect calls for ELSI work to bridge science, law, policy, and publics when new science becomes socially relevant (Greenbaum, 2013).

Recommendations to increase accessibility and responsiveness in OSPW remediation include the following. Communication issues could be addressed through the community-inclusive development of a common language dictionary about OSPW remediation, particularly CTWS enhanced by genomics. Affected communities should be made aware of OSPW remediation methods and consequences early in decision making timelines, in plain languages spoken by those affected by the remediation, and with care to the presentation of terminology and context. Knowledge sharing opportunities and support from scientists and regulators should also be made available, as should opportunities for researchers, industry representatives, and decision makers to learn community members’ cultural worldviews and viewpoints.

While the participants in the Indigenous Peoples session stressed that more members of Treaty Eight directly affected by oil sands development should have the loudest voice in this conversation, this raised the question of who is responsible for ensuring that members of Treaty Eight have total capacity to engage. With sufficient capacity and communication in various modalities, Indigenous Peoples from Treaty Eight could take the lead on future remediation work.

This study confirms that engagement with persons affected by oil sands remediation is particularly challenging, with obstacles that include oil spills, tailings pond seepage, and COVID-19, technical uncertainty, histories of mistrust, and uneven capacity to participate. Effective engagement therefore requires time, resources, plain-language and culturally responsive communication, early involvement in decision-making, and explicit attention to the ethical, legal, social, and cultural dimensions of genomics-enhanced remediation. For CTWS enhanced by genomics, meaningful participation cannot begin after technical decisions are already stabilized; it must shape how technologies are defined, tested, monitored, governed, and judged acceptable. The central contribution of this study is therefore to show that affected persons’ perspectives are not barriers to genomically-informed or enhanced remediation innovation; instead, they are evidence needed to design remediation systems that are technically credible, socially accountable, legally attentive, and culturally responsive.

## Data Availability

The raw data supporting the conclusions of this article will be made available by the authors, without undue reservation.
